# Identification of Key Proteins and Networks Related to Grain Development in Wheat (*Triticum aestivum* L.) by Comparative Transcription and Proteomic Analysis of Allelic Variants in *TaGW2-6A*

**DOI:** 10.3389/fpls.2016.00922

**Published:** 2016-06-28

**Authors:** Dengfeng Du, Xin Gao, Juan Geng, Qingyan Li, Liqun Li, Qian Lv, Xuejun Li

**Affiliations:** State Key Laboratory of Crop Stress Biology in Arid Areas and College of Agronomy, Northwest A&F UniversityYangling, China

**Keywords:** grain development, proteomic, *TaGW2-6A*, two-dimensional gel electrophoresis, wheat

## Abstract

In wheat, coding region allelic variants of *TaGW2-6A* are closely associated with grain width and weight, but the genetic mechanisms involved remain unclear. Thus, to obtain insights into the key functions regulated by *TaGW2-6A* during wheat grain development, we performed transcriptional and proteomic analyses of *TaGW2-6A* allelic variants. The transcription results showed that the *TaGW2-6A* allelic variants differed significantly by several orders of magnitude. Each allelic variant of *TaGW2-6A* reached its first transcription peak at 6 days after anthesis (DAA), but the insertion type *TaGW2-6A* allelic variant reached its second peak earlier than the normal type, i.e., at 12 DAA rather than 20 DAA. In total, we identified 228 differentially accumulated protein spots representing 138 unique proteins by two-dimensional gel electrophoresis and tandem MALDI-TOF/TOF-MS in these three stages. Based on the results, we found some key proteins that are closely related to wheat grain development. The results of this analysis improve our understanding of the genetic mechanisms related to *TaGW2-6A* during wheat grain development as well as providing insights into the biological processes involved in seed formation.

## Introduction

Wheat (*Triticum aestivum* L., 2*n* = 6*x* = 42, AABBDD) is an allohexaploid species and one of the most important and widely cultivated crops throughout the world. However, due to increases in the global population and decreases in cultivatable land, current wheat production levels cannot meet the needs of the world's population (Flachsbarth et al., 2015; Wei et al., 2015). A valid approach to address this problem involves cloning and exploiting genes related to grain weight, thereby improving yields (Barrero et al., 2011). Elucidating the interaction networks of these genes will help to understand the molecular mechanism of seed formation.

*TaGW2* is a grain weight-related gene and an orthologous gene of *OSGW2* (Su et al., 2011), which encodes a functional E3 RING ubiquitin ligase that exhibits nucleocytoplasmic subcellular partitioning. Recently, several studies have examined the effects of a homologous gene in wheat (*TaGW2-6A*) on the grain size parameters. Several single nucleotide polymorphisms (SNPs) were found in its promoter region, which have significant associations with the kernel width (KW) and thousand kernel weight (TKW; Su et al., 2011; Zhang et al., 2013; Jaiswal et al., 2015). Further transcription analysis has shown that *TaGW2-6A* is negatively associated with grain width (Yang et al., 2012; Hong et al., 2014; Jaiswal et al., 2015). Interestingly, a single-base insertion in the eighth exon of *TaGW2-6A* causes premature termination in a large-kernel variety (Lankaodali), which ultimately leads to increases in the grain width and weight (Yang et al., 2012).

The ubiquitin-proteasome system (UPS) plays an important role in the determination of seed size and stress tolerance in plants (Santner and Estelle, 2010; Capron et al., 2012). Numerous proteins are involved in wheat grain development and maintenance (Ma et al., 2014), where this dynamic process comprises two main stages. The first stage involves the early and rapid division of the zygote and triploid nucleus, which occurs at approximately 3–20 days after anthesis (DAA), where this process is mainly related to grain enlargement. During the second stage, cell division slows and then ceases from around 10 DAA until maturity, when the grain starts to fill, storage products accumulate, and the endosperm then functions as a carbohydrate store (Nadaud et al., 2015).

Two-dimensional gel electrophoresis (2-DE) and mass spectrometry (MS) proteomic approaches have been employed widely to investigate the expression profiles of proteins related to grain dynamics in different species, including Arabidopsis (Aryal et al., 2015), rice (Liao et al., 2014), and maize (Guo et al., 2013). In recent years, several proteomic studies have investigated wheat grain development, where Zhang et al. (2015) identified some key proteins that are closely related to grain size and abiotic stress adaptation by analyzing the proteomic differences in the middle and late stages of wheat grain development. A proteomics analysis of wheat seed germination also detected modifications in phosphorylation that can enhance environmental stress defenses in wheat (Dong et al., 2015). Moreover, an analysis of the proteins that respond to heat stress during grain filling indicated that some of the key enzymes involved in photosynthesis may contribute to higher heat tolerance (Wang et al., 2015b). According to a proteomic dynamics analysis of albumins and globulins during grain development, 12 genes exhibit differential expression during the transcriptional and translational stages (Dong et al., 2012). Furthermore, proteomic characterization of five phases during wheat grain development also identified some storage proteins that are related to flour quality (Guo et al., 2012).

Near-isogenic lines (NILs) comprise a set of materials with extremely similar backgrounds except for the target gene. NILs have been employed widely as important tools in molecular marker-assisted breeding in Arabidopsis (Melchinger et al., 2007), rice (Moumeni et al., 2015), and maize (Wang et al., 2015a), thereby determining the effects of the target gene based on comparisons of the proteomic characteristics. Thus, hard and soft wheat NILs were used to analyze the proteome during four grain development stages, where the results indicated that the kernel hardness is related to the amplification of a stress response during endosperm development (Lesage et al., 2011). In addition, the antioxidant system is significantly inhibited in the flag leaf of an early aging wheat line compared with its normal aging NIL (Li et al., 2014).

The Gene Ontology (GO) is a community-based bioinformatics resource, which provides information about gene product functions by using ontologies to represent biological knowledge. The GO project is a comprehensive resource for functional genomics, which provides evidence-supported annotations that describe the biological roles of individual genomic products (Blake et al., 2015). The Kyoto Encyclopedia of Genes and Genomes (KEGG) pathway maps are also used widely for the biological interpretation of genome sequences and other types of high-throughput data (Kanehisa et al., 2014). The GO and KEGG pathway enrichment are basic tools that are used in the proteomic analysis in plants, such as Arabidopsis (Mostafa et al., 2016), rice (Li et al., 2016), and wheat (Yang et al., 2016).

Most previous studies of *TaGW2-6A* have focused on its function, whereas the mechanisms that underlie its roles during grain development still unknown at the proteomic level. In the present study, we used a NIL to analyze the transcription abundance and dynamic proteome characteristics of *TaGW2-6A* allelic variants during wheat grain development. We employed the GO and KEGG to identify the key proteins and to construct their networks. Our results give insights into the functions of allelic variants of TaGW2-6A during wheat grain development as well as providing valuable information that helps to elucidate the molecular and genetic basis of grain size and kernel weight in bread wheat.

## Materials and methods

### Plant materials

The BC_6_F_2_ population were derived from a cross between the Chinese winter wheat cultivar Lankaodali (LK, TKW = 57.49 ± 0.88 g, with the insertion of a T nucleotide base at the 977-bp position in the eighth exon of the *TaGW2* allele compared with Chinese Spring) and Chinese Spring (CS, TKW = 27.75 ± 0.62 g), where recurrent backcrossing with the parent CS was performed for six generations, which was accompanied by marker-assisted selection with SNPs (Yang et al., 2012) to allow the rapid and efficient recovery of the recurrent parent genome. One BC_6_F_2_ plant was self-pollinated for four generations to obtain the NIL-31 according to its larger kernel size, higher grain weight phenotype, and the T nucleotide base insertion in the *TaGW2* coding sequence genotype. The full sequence of *TaG*W2 and its amino acid from CS, NIL-31, and LK are shown in Tables S1, S2. A previous mapping assay located the *TaGW2* mutation allele on chromosome 6A (Yang et al., 2012).

NIL-31 and CS were planted at Northwest Agriculture and Forestry University (longitude 108°4′E, latitude 34°16′N) in China during the cropping seasons in 2014–2015 under non-stressed natural soil conditions, where 30 seeds per family were individually planted manually with eight lines at 25 cm apart per 2 m row, with a line spacing of 15 cm, and the field plots were managed according to the same methods employed for commercial production. The main stem ears were marked with different color tags in the morning when the anthers first appeared outside the florets of the spikelets. The labeled spikelets were harvested from NIL-31 and CS at seven different sampling dates, i.e., 3, 6, 9, 12, 15, 20, and 25 DAA. The grain tissues from the middle part of the spike were separated and isolated, snap frozen, and then stored at −80°C.

### Measurement of grain traits

Grain samples were collected at each development stage and the kernel length (KL) and KW were measured in 30 randomly selected kernels. Three independent fresh samples comprising 200 kernels were weighed and converted into the TKW for the final data analysis. Statistical analyses were conducted using SPSS 17.0 for Windows (SPSS Inc., Chicago, IL).

### RNA extraction and cDNA synthesis

Total RNA was extracted from NIL-31 and CS seeds at different stages using TRIzol reagent, according to the manufacturer's instructions (TaKaRa Biotechnology, Dalian, China). The concentration and quality of the RNA samples were determined using a nucleic acid and protein analysis system (Biophotometer Plus, Eppendorf, Germany). The first-strand cDNA was synthesized using 2 μg purified RNA, AMV reverse transcriptase, and Oligo (dT15) primers, according to the manufacturer's instructions (TaKaRa, Japan).

### Transcription analysis of *TaGW2-6A* allelic variants

Real-time quantitative reverse transcription-PCR (Q-RT-PCR) and RT-PCR were conducted to analyze the transcript levels of *TaGW2-6A* allelic variants. Q-RT-PCR was performed with Faststart Essential DNA Green Master (Roche, Germany) using a LightCycler^®^ 96 detection system (Roche, Switzerland) according to the manufacturer's instructions. The PCR reaction mixture for *TaGW2-6A* comprised a total volume of 12.5 μL, which contained 100 ng cDNA, 6.25 μL of 2X Faststart Essential DNA Green Master (Roche, Germany), 0.45 μL of each primer (4.5 μM), and 4.1 μL of double-distilled H_2_O (ddH_2_O). Q-RT-PCR was performed using the following thermal profile: 95°C for 5 min, 40 cycles at 95°C for 30 s, and 60.4°C for 30 s, followed by 72°C for 10 min. The relative *TaGW2-6A* transcript levels were calculated using the *2*^−Δ*ΔCT*^ method (Livak and Schmittgen, 2001), where the bread wheat *Actin* gene (18S) was used as an internal reference. Three biological replicates were performed for each allelic variant and three technical replicates were analyzed for each biological replicate, where the 3 DAA stage of CS was used as a reference sample for ΔΔ*Ct*. The *TaGW2-6A* primer sequences were as follows (Qin et al., 2014a): forward, 5′-CTGCGGAAAGTTCACCAGAT AG-3′; and reverse, 5′-TGTCAGCAAAAGGCAACGGTA-3′. The *18S* primer sequences were as follows: forward, 5′-CG TCCCTGCCCTTTGTACAC-3′; and reverse, 5′-AACACT TCACCGGACCATTCA-3′. All of the primers used in this study were obtained from AuGCT (Beijing).

### Protein preparation

Grain proteins were extracted according to the following procedures. First, 900 mg grain samples were ground rapidly into a fine power in liquid nitrogen using a mortar and pestle. The homogenate was precipitated overnight with 10 mL of extraction buffer containing 10% trichloroacetic acid/acetone, 0.2% dithiothreitol (DTT), at −20°C, before centrifugation at 16,000 × g for 30 min at 4°C, and the supernatant was removed. The pellet was rinsed with 100 mM ammonium acetate in 80% methanol. After centrifugation at 16,000 × g for 30 min at 4°C, the supernatant was removed and the pellet was rinsed with ice-cold 80% acetone. Following another round of centrifugation at 16,000 × g for 30 min at 4°C, the supernatant was removed and the pellet was air-dried for 10 min in a fume cupboard. The pellet was then resuspended in 10 mL phenol/sodium dodecyl sulfate (SDS) extraction buffer, before adding Tris-saturated phenol (pH 8.0):SDS buffer solution (30% sucrose, 2% SDS, 5% β-mercaptoethanol) at 1:1 for 5 min. Following centrifugation at 6000 × g for 3 min at 4°C, the phenolic phase was collected and precipitated overnight with five times the volume of 100 mM ammonium acetate in methanol at −20°C. After centrifugation at 16,000 × g for 10 min at 4°C, the supernatant was removed and the precipitate was rinsed twice with methyl alcohol and 80% acetone. The pellet was air-dried, resuspended in 300 mL of lysis buffer containing 7 M urea, 2 M thiourea, 4% 3-[(3-cholamidopropyl) dimethylammonio]-1-propanesulfonate (CHAPS), 65 mM DTT, and 0.2% (w/v) Bio-Lyte, and then vortexed for 1 h at room temperature. The protein concentration was determined using the Bradford assay (Bio-Rad) based on a bovine serum albumin standard (Li et al., 2013a). The detailed standard curve obtained by the Bradford assay for samples resuspended in ddH_2_O is shown in Table S3.

### 2-DE and image analysis

For 2-DE, 900 μg protein samples were loaded onto a Ready Strip TMIPG Strip (17 cm, pH 4–7, Bio-Rad, USA) and hydrated passively with 450 μL of protein solution containing 0.5% (v/v) immobilized pH gradient buffer (pH 4–7) for 12–16 h at 20°C using a Protean IEF Cell (Bio-Rad, USA).

First dimension isoelectric focusing (IEF) was performed in six steps: 250 V for 60 min, 250 V for 90 min, 500 V for 90 min, 1000 V for 2 h, 8500 V for 5 h, and 8500 V for 10 h, with a total of 85 kV and a constant voltage of 500 V in the last 24 h. After IEF, the strips were incubated for 15 min in “equilibration buffer I” comprising 6 M urea, 2% (w/v) SDS, 1.5 M Tris-HCl (pH 8.8), 20% (v/v) glycerol, 0.01% (w/v) bromophenol blue, and 2% (w/v) DTT, and then for 15 min in “buffer II” comprising 6 M urea, 2% (w/v) SDS, 1.5 M Tris-HCl (pH 8.8), 20% (v/v) glycerol, 0.01% (w/v) bromophenol blue, and 2.5% (w/v) iodoacetamide.

For electrophoresis in the second dimension, the strips were transferred to 11.7% vertical SDS-polyacrylamide gel electrophoresis (SDS-PAGE) gels. All of the grain samples were run in triplicate to obtain statistically reliable results. After electrophoresis, the gels were fixed in 40% (v/v) methanol and 10% (v/v) acetic acid for 40 min. To visualize the gels, they were stained with staining solution comprising 0.12% (v/v) Coomassie Brilliant Blue G-250, 20% (v/v) alcohol, 10% (v/v) phosphoric acid, and 10% (w/v) ammonium sulfate, before destaining in ddH_2_O (Wang et al., 2012). The 2-DE images were scanned at 300 dpi with a UMAX Power Look 2100XL scanner (Maximum Tech, Taiwan, China) and quantitative intensity analysis was performed using PDQuest (Version 8.0.1, Bio-Rad, USA). Three technical replicates were analyzed for each developmental stage.

The 2-DE gels for the 6-DAA seed samples of CS and NIL-31 were selected initially as the reference gels. Two other stages (12 and 20 DAA) were matched to the reference gels. Groups were formed automatically and single spots that differed between replicates were checked manually and corrected when necessary. The spots present in three independent sample sets were selected. Quantitative image analysis detected significant differences in the abundance of protein spots using the Student's *t*-test (difference in abundance of at least two times, *P* < 0.05).

### Identification of differentially expressed proteins (DEPs) and matrix-assisted laser desorption/ionization time-of-flight MS (MALDI-TOF/TOF-MS) analysis

The DEPs in three grain stages, i.e., 6, 12, and 20 DAA, were identified between CS and NIL-31 using PDQuest. The standards for the DEPs were obtained as described in a previous study (Guo et al., 2012). The DEPs were manually excised from gels using free enzyme tips, washed three times with Milli-Q water, destained with 100 mM NH_4_HCO_3_, dried twice with 100% acetonitrile, and each spot sample was digested overnight at 37°C by adding 5 μL sequencing grade modified trypsin (Promega, Madison, WI, USA)/NH_4_HCO_3_ (50 mM) solution at a concentration of 10 ng/μL. The peptides were extracted twice with 0.1% trifluoroacetic acid (TFA) in 50% acetonitrile. The extracts were pooled and lyophilized. The lyophilized tryptic peptides were then dissolved in 5 mg/mL α-cyano-4-hydroxycinnamic acid containing 0.1% TFA and 50% acetonitrile. MALDI-TOF/TOF-MS analyses were conducted using an AB 4800 system (Applied Biosystems). The mass range was 700–3500 in the positive reflection mode for peptide mass fingerprints and 40–1015 for LIFT. All of the spectra obtained for proteins were searched against wheat databases on the internet using the MASCOT search engine. The search parameters were: 100 ppm mass tolerance for peptides and 0.5 Da mass tolerance for TOF/TOF fragments, cysteines carbamidomethylation as a fixed modification, and methionine oxidation as a variable modification. The confidence in the peptide mass fingerprinting matches (*P* < 0.05) was based on the MOWSE score and confirmed by the accurate overlapping of the matched peptides with the major peaks of the mass spectrum. Only significant hits were accepted, as defined by the MASCOT probability analysis (*P* < 0.05).

### GO and KEGG pathway enrichment analysis

To perform the GO and KEGG pathway enrichment analyses, homology searches were first performed for all of the query protein matches using BLASTp against the *Arabidopsis thaliana* protein database. The *E*-value was set to 1e-10 and the top 10 best hits were taken for each query sequence. Among the 10 best hits, the hit with the highest shared identity with the query was selected as homologous. The GO analysis was performed with different mapping steps to link all of the BLAST hits with the functional information stored in the GO database using the DAVID toolkit. Public resources such as NCBI, PIR, and GO were used to create links with protein IDs and the corresponding GO information. All of the annotations had associated evidence codes, which provided information regarding the quality of the functional assignments. For the KEGG pathway enrichment analysis, we performed pathway enrichment using annotated proteins in the query dataset against the KEGG database. To better understand the functions and interactions of identified proteins, a protein–protein interaction network (PPI) was predicted. The protein identifications were based on different organisms listed in the NCBI wheat database, so all of the identified proteins were BLASTed against the *Arabidopsis thaliana* protein database (http://www.arabidopsis.org) to obtain annotated protein entries for PPI tools. A PPI was constructed with the online analysis tool STRING 9.0 as follows: protein accessions were submitted to http://string-db.org and the results with the highest score and lowest *E*-value were considered to be relevant for each protein identified.

## Results

### Comparison of grain size in CS and NIL-31

**Figure 1:**
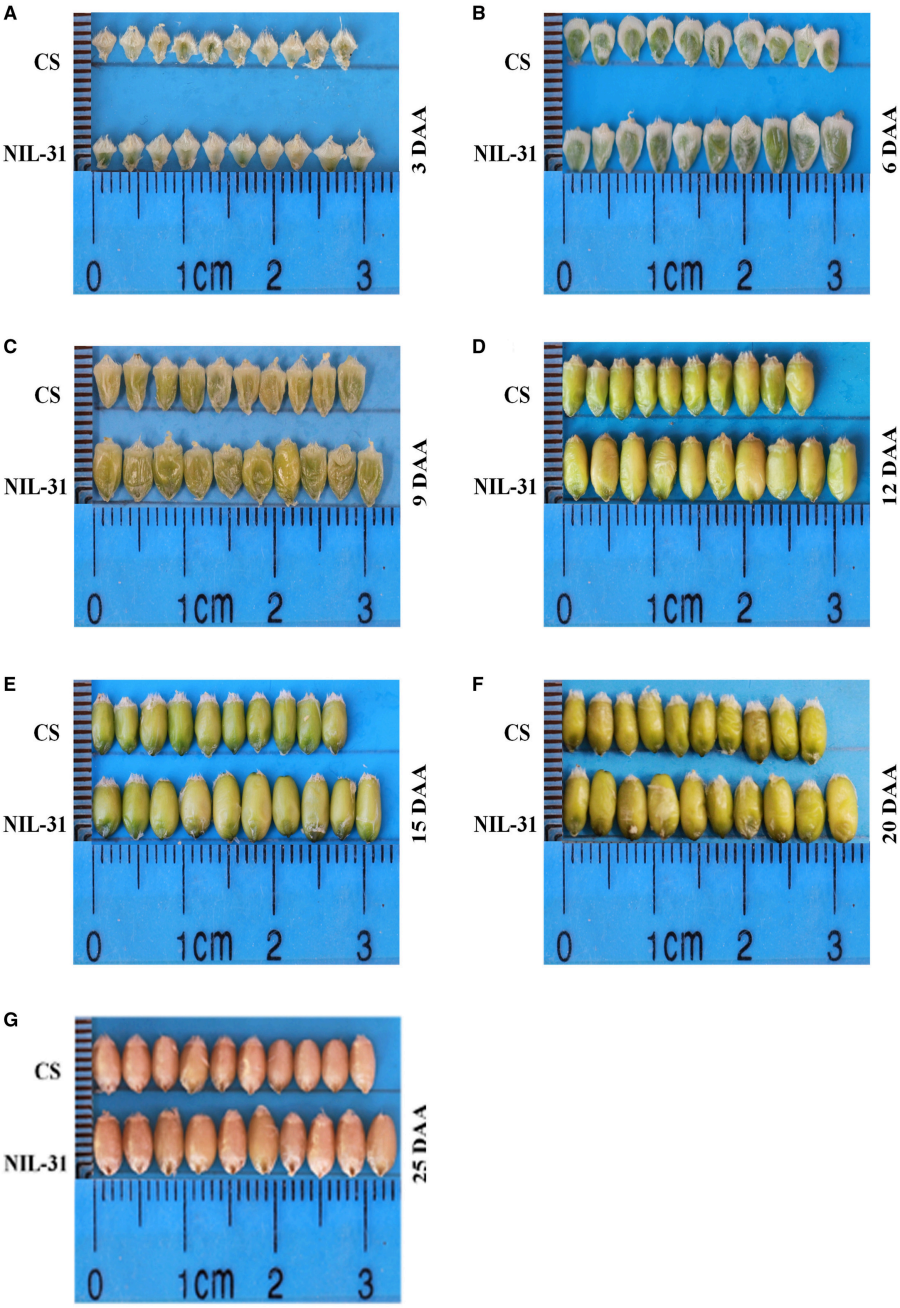
**Grain development at seven stages in Chinese Spring and NIL-31. (A)** 3 days after anthesis (DAA); **(B)** 6 DAA; **(C)** 9 DAA; **(D)** 12 DAA; **(E)** 15 DAA; **(F)** 20 DAA; **(G)** 25 DAA.

**Figure 2:**
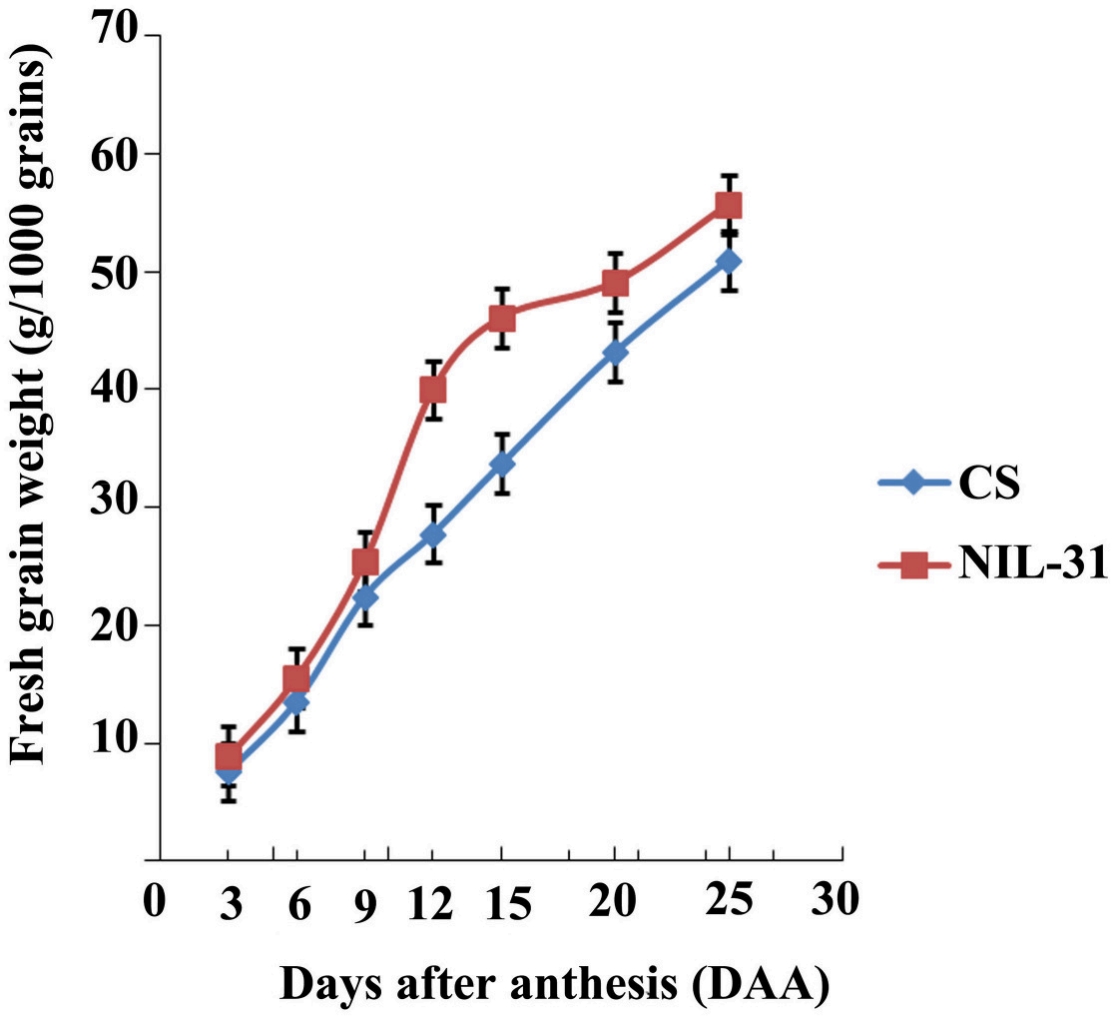
**Fresh grain weight accumulation in Chinese Spring and NIL-31**.

The grain size and weight increased gradually from 3 to 25 DAA in both CS and NIL-31 (Figures 1A–G). However, NIL-31 had a higher biomass accumulation rate than CS, especially from 9 to 15 DAA (Figure 2). Either KL or TKW differed significantly (*P* ≤ 0.01) between CS and NIL-31 in all seven grain development stages. Excluding 3 DAA, KW differed significantly between CS and NIL-31 in the other six stages. The detailed results are shown in Table 1. These results showed that the insertion type allelic variant of *TaGW2-6A* was closely related to the grain size and kernel weight.

**Table 1:** **Grain traits of CS and NIL-31 at different days after anthesis (DAA)**.

**Trait**	**Material**	**Grain development stage**						
		**3 DAA**	**6 DAA**	**9 DAA**	**12 DAA**	**15 DAA**	**20 DAA**	**25 DAA**
KL (mm)	CS	3.00 ± 0.17Aa	3.76 ± 0.53Aa	5.03 ± 0.18Aa	6.17 ± 0.33Aa	6.33 ± 0.25Aa	6.50 ± 0.09Aa	6.83 ± 0.30Aa
	NIL-31	3.23 ± 0.10Bb	4.93 ± 0.18Bb	6.00 ± 0.57Bb	6.70 ± 0.23Bb	6.70 ± 0.23Bb	7.30 ± 0.38Bb	7.47 ± 0.05Bb
KW (mm)	CS	2.23 ± 0.14Aa	2.87 ± 0.13Aa	2.90 ± 0.09Aa	3.15 ± 0.22Aa	3.08 ± 0.14Aa	3.07 ± 0.12Aa	3.13 ± 0.22Aa
	NIL-31	2.33 ± 0.11Aa	2.89 ± 0.14Bb	3.00 ± 0.08Bb	3.16 ± 0.22Bb	3.20 ± 0.19Bb	3.17 ± 0.22Bb	3.73 ± 0.18Bb
TKW (g)	CS	7.76 ± 0.17Aa	12.25 ± 0.20Aa	24.24 ± 0.11Aa	27.77 ± 0.13Aa	32.24 ± 0.09Aa	43.69 ± 0.12Aa	50.28 ± 0.09Aa
	NIL-31	8.12 ± 0.36Bb	17.00 ± 0.53Bb	26.22 ± 0.50Bb	40.30 ± 0.06Bb	40.30 ± 0.06Bb	50.35 ± 0.03Bb	55.00 ± 0.02Bb

*All of the data represent the mean ± SE. A and B indicate differences detected using Duncan's test at P ≤ 0.01. a and b indicate differences detected using Duncan's test at P ≤ 0.05 (starting from a, b is significantly different from a). KL, kernel length; KW, kernel weight; TKW, thousand kernel weight*.

### Transcription levels in *TaGW-6A* allelic variants

**Figure 3:**
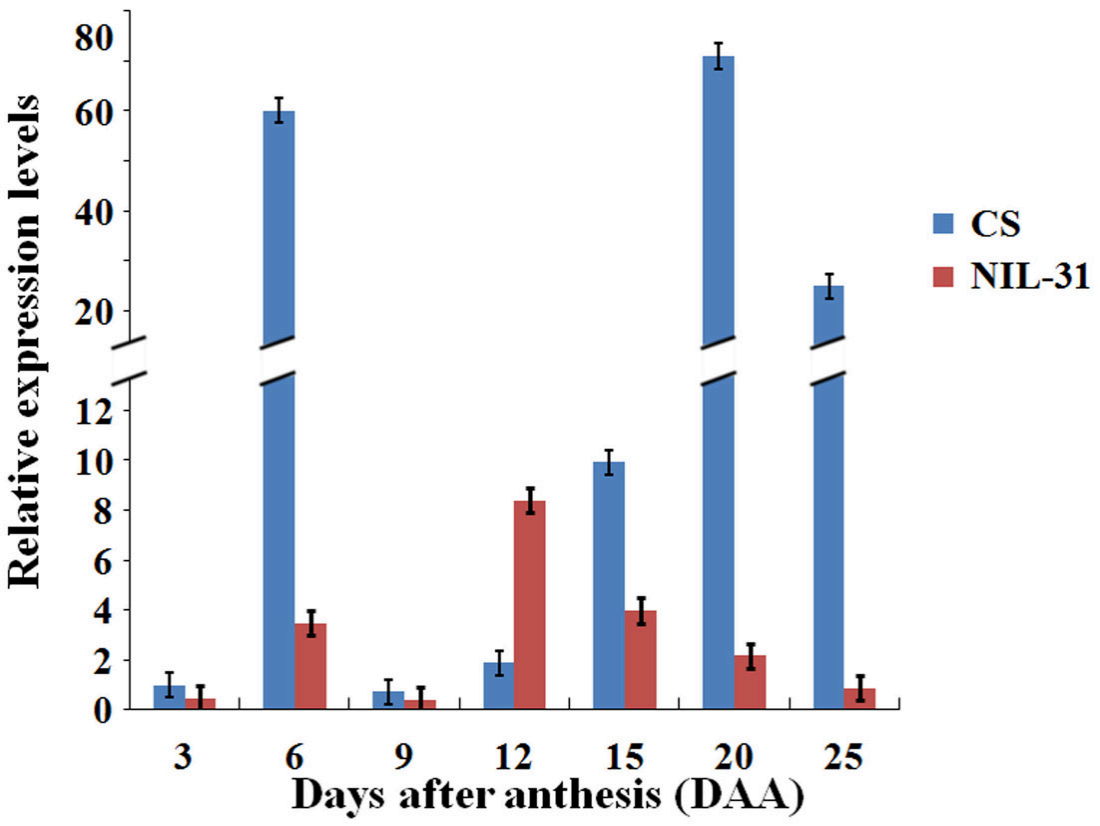
**Mean relative expression levels of ***TaGW2-6A*** at different stages of grain development in Chinese Spring and NIL-31**.

The average expression levels of *TaGW-6A* in CS and NIL-31 during the seven development stages are shown in Figure 3. Clearly, the transcription abundance levels of the *TaGW2-6A* allelic variants differed significantly by several magnitudes. During the seven grain development stages, the transcription patterns of both *TaGW2-6A* allelic variants had two expression peaks. The first peak always occurred at 6 DAA, but the second peak was reached earlier by the insertion type than the normal type, i.e., at 12 DAA rather than 20 DAA. We compared the transcription levels in CS and NIL-31, and the two stages with the greatest differences were 6 DAA and 20 DAA. Interestingly, the expression level of the insertion type was only slightly higher than the normal type at 12 DAA, whereas the expression patterns differed clearly at the other stages.

### Analysis of DEPs in CS and NIL-31

Based on the transcription results, we performed proteomic analyses between CS and NIL-31 using 2-DE and MALDI-TOF/TOF-MS at 6, 12, and 20 DAA to obtain insights into the functions of the encoded proteins based on their network interactions, as well as elucidating the regulatory mechanisms related to *TaGW2-6A*.

**Figure 4:**
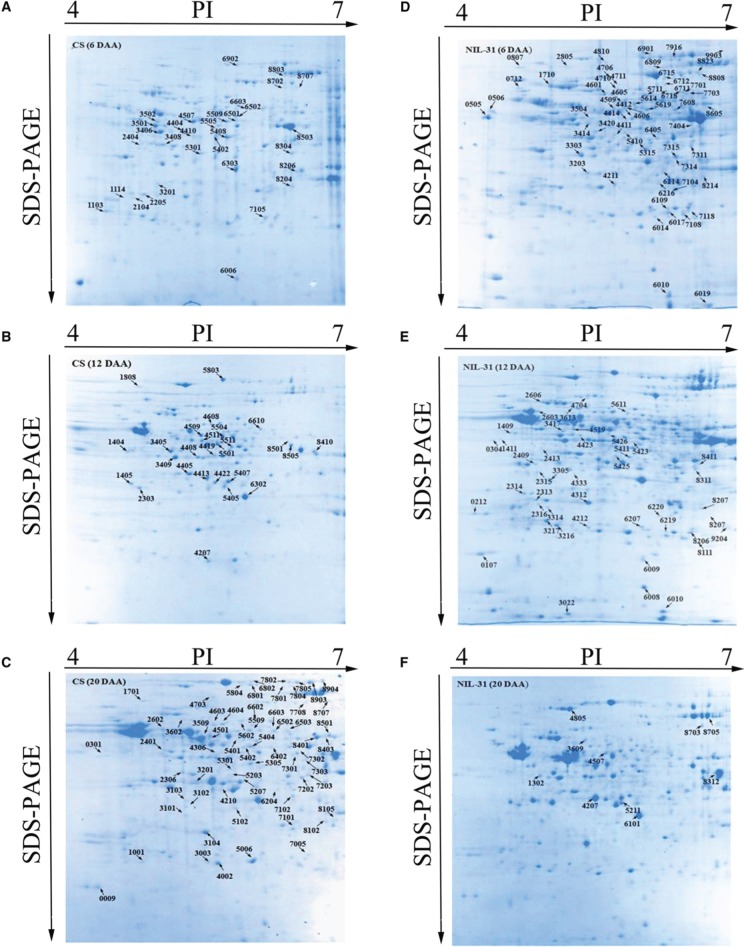
**2-DE maps of proteins extracted from the Chinese Spring and NIL-31 samples. (A–C)** 2-DE maps during three grain development at 6 days after anthesis (DAA), 12 DAA, and 20 DAA, in Chinese spring, respectively; **(D–F)** 2-DE maps during three grain development stages at 6 DAA, 12 DAA, and 20 DAA in NIL-31, respectively.

CS and NIL-31 had similar proteomic profiles at the three stages according to the representative gel images for both samples. In total, 228 protein spots were identified as DEPs between CS and NIL-31 at three stages, i.e., 6, 12, and 20 DAA. There were 48 upregulated protein spots in NIL-31, including 22 spots at 6 DAA, 19 spots at 12 DAA, and seven spots at 20 DAA. In total, 67 protein spots were downregulated in NIL-31, including 20 spots at 6 DAA, 19 spots at 12 DAA, and 28 spots at 20 DAA. Overall, 63 protein spots exhibited qualitative changes in NIL-31, including 33 spots at 6 DAA, 27 spots at 12 DAA, and three spots at 20 DAA, as well as 50 in CS, including 12 spots at 6 DAA, seven spots at12 DAA, and 31 spots at 20 DAA. All of the DEPs are marked by their spot numbers in the gel images shown in Figures 4A–F.

### Identification and functional classification of DEPs

Among the 228 spots identified, 88 had functional annotations in the database. We identified the other 140 uncharacterized proteins or hypothetical proteins by submitting their sequences as queries to search for homologs using BLASTP (UniProt). The corresponding best homologs shared at least 80% sequence similarity, thereby suggesting that they might possess similar functions. We found that the 228 identified spots represented 138 unique proteins. Detailed information about the protein spots is provided in Table S4 and Data Sheet S1.

**Figure 5:**
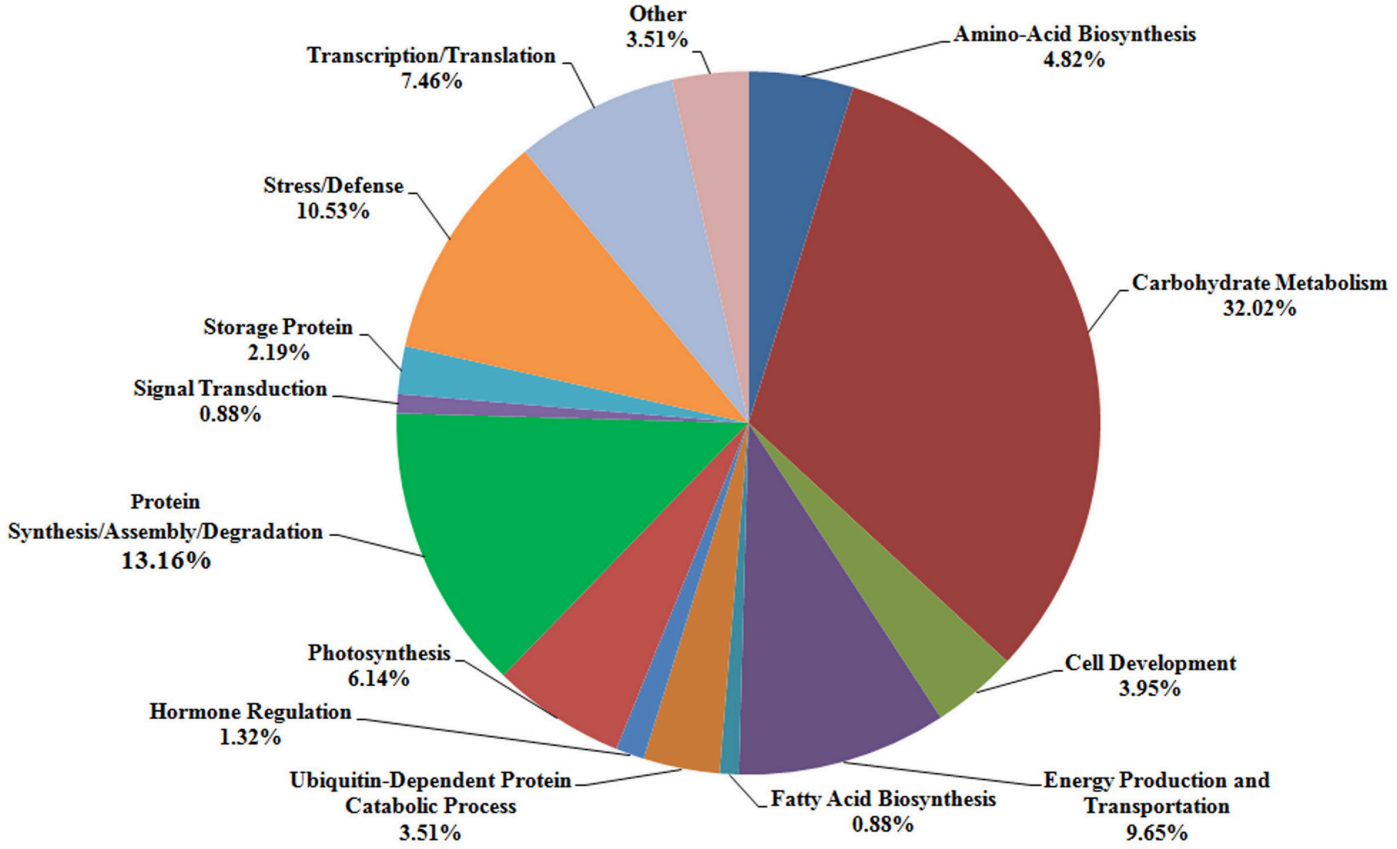
**Distribution of the proteins identified at three grain development stages in Chinese Spring and NIL-31**. Fourteen protein groups were categorized based on their putative functions.

According to the different functions, the 228 identified protein spots were classified into 14 groups, i.e., amino-acid biosynthesis (4.82%), carbohydrate metabolism (32.02%), cell development (3.95%), energy production and transportation (9.65%), fatty acid biosynthesis (0.88%), ubiquitin-dependent protein catabolic process (3.51%), hormone regulation (1.32%), photosynthesis (6.14%), protein synthesis/assembly/degradation (13.16%), signal transduction (0.88%), storage protein (2.19%), stress/defense (10.53%), transcription/translation (7.46%), and other unknown function groups (3.51%), as shown in Figure 5. The proteins associated with carbohydrate metabolism belonged to five sub-categories: (1) starch metabolism (6.14%, 14 proteins), (2) glycolysis (13.60%, 31 proteins), (3) nitrogen metabolism (0.44%, one protein), (4) the tricarboxylic acid (TCA) pathway (3.51%, eight proteins), and (5) carbohydrate metabolism (8.33%, 19 proteins).

### GO and KEGG analysis of the DEPs

**Figure 6:**
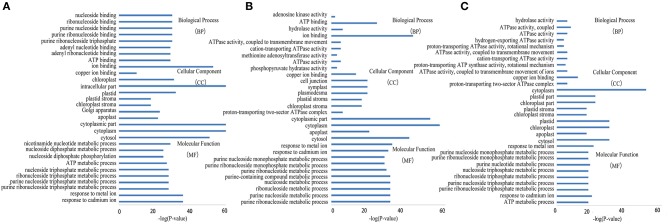
**GO enrichment analysis of differentially expressed proteins at each developmental stage. (A)** 6 days after anthesis (DAA); **(B)** 12 DAA; **(C)** 20 DAA.

**Figure 7:**
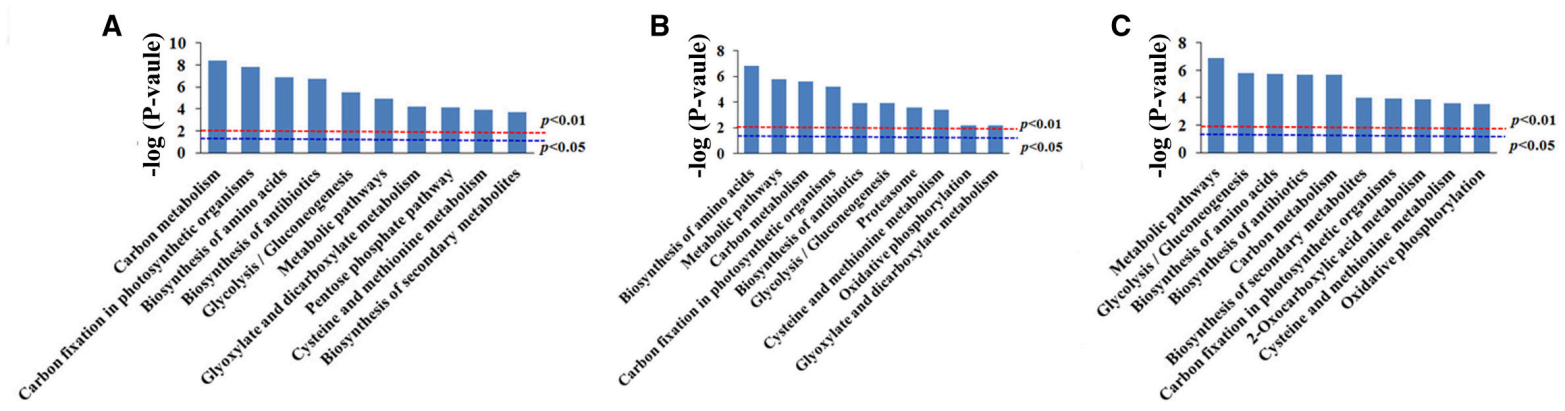
**KEGG enrichment analysis of differentially expressed proteins at each developmental stage. (A)** 6 days after anthesis (DAA); **(B)** 12 DAA; **(C)** 20 DAA.

**Figure 8:**
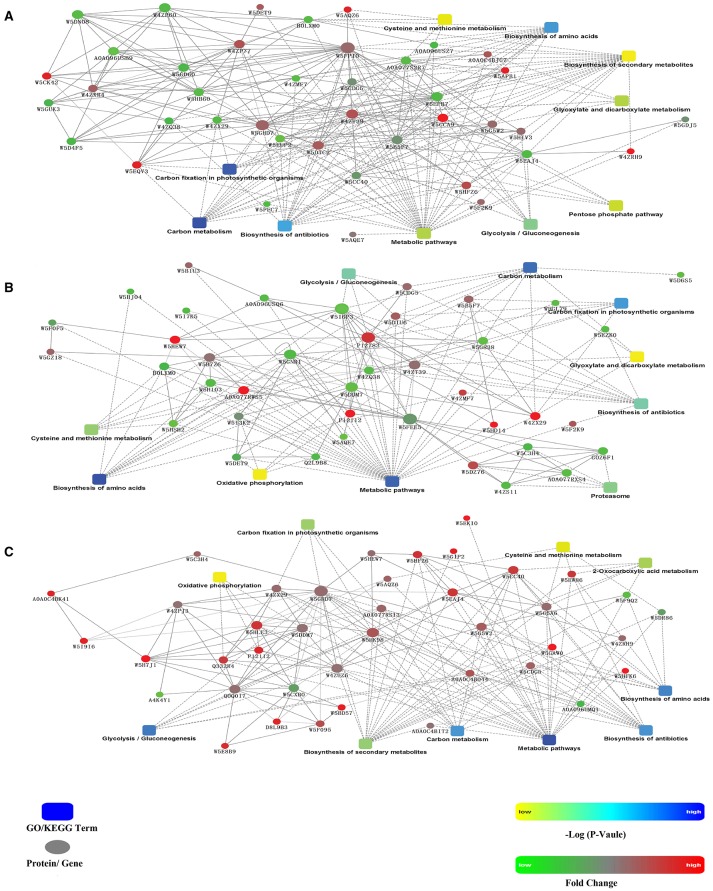
**Protein–protein interactions obtained based on the analysis using the STRING database. (A)** 6 days after anthesis (DAA); **(B)** 12 DAA; **(C)** 20 DAA.

GO enrichment was performed to investigate the protein expression profiles. The results demonstrated that 176 (68 spots at 6 DAA, 56 spots at 12 DAA, and 52 spots at 20 DAA), 167 (66 spots at 6 DAA, 51 spots at 12 DAA, and 50 spots at 20 DAA), and 173 (68 spots at 6 DAA, 55 spots at 12 DAA, and 50 spots at 20 DAA) protein categories were enriched in the Biological Process (BP), Cellular Component (CC), and Molecular Function (MF) categories. In particular, response to cadmium ion, cytosol, and ion binding were the most common in the BP, CC, and MF categories, respectively. The DEPs were predominantly related to ion binding in organic substance biosynthetic processes at 6 DAA. At 12 DAA, the DEPs were related mainly to copper ion binding involving organic substance biosynthetic processes. At 20 DAA, the DEPs were generally proteins related to small molecule binding and the regulation of nucleotide binding and nucleoside phosphate binding (Figure 6). Further KEGG pathway enrichment showed that the DEPs were mainly involved in carbon metabolism at 6 DAA, biosynthesis of amino acids at 12 DAA, and metabolic pathways at 20 DAA (Figure 7). PPI analysis further indicated the presence of a diversified functional network comprising these proteins at different developmental stages (Figure 8). Detailed information is provided in Table S5 and Data Sheet S2.

Among the DEPs, some spots were expected to be the same protein (Table S4). However, the GO and KEGG analyses showed that these DEPs belonged to different biochemical process groups during wheat grain development. For example, spots 4601 (6 DAA), 4608 (12 DAA), 4603 (20 DAA), and 4604 (20 DAA) shared the same protein name, i.e., 2,3-bisphosphoglycerate-independent phosphoglycerate mutase (iPGM), but protein spot 4601 (6 DAA) was classified as belonging to the response to cadmium ion group, whereas protein spot 4608 (12 DAA) was assigned to purine ribonucleoside metabolic process. Protein spots 4603 and 4604 (20 DAA) were categorized as belonging to ATP metabolic processes. These isoforms are probably attributable to protein phosphorylation. Several other groups are shown in Table S6.

## Discussion

Our results demonstrate that *TaGW2-6A* is a negative regulator of grain size and weight, which is consistent with previous reports (Yang et al., 2012; Hong et al., 2014; Jaiswal et al., 2015). Moreover, the formation of the characteristic endosperm structure is followed by a sequence of cell division, expansion, and differentiation, where the main differentiation period starts at 6 DAA and lasts for about 5 days (Wan et al., 2008). This start point agrees with the first high abundance level of each allelic variant of *TaGW2-6A*. The rapid grain-filling stage usually lasts for 10 days from 12 and 21 DAA (Wan et al., 2008), which agrees with the second high abundance level in the insertion mutant type, and the level was higher than that in the normal type. Therefore, we suggest that *TaGW2-6A* has an essential role in regulating cell differentiation and grain filling.

Plants utilize the UPS to modulate most aspects of growth and development (Santner and Estelle, 2010), and several factors that are involved with ubiquitin-related activities have recently been shown to determine seed size in Arabidopsis (Li and Li, 2014) and rice (Song et al., 2007). E3s are prerequisites for ubiquitination, which ensure the high specificity and selectivity of UPS (Glickman and Ciechanover, 2002).

*TaGW2* encodes a functional E3 RING ubiquitin ligase with nucleocytoplasmic subcellular partitioning (Su et al., 2011). However, the *TaGW2-6A* insertion mutation reduces the coding protein sequence from the usual 424 amino acids (47.2 kDa) to 328 amino acids (37.1 kDa), i.e., a truncation of 96 amino acids (Yang et al., 2012). The function of this mutant protein differs from that of the normal protein during metabolic processes.

Among the proteins identified in this study, four DEPs (spots 6207, 3003, 4212, and 9204) were identified as proteasome subunit alpha type subunits (Table S4). The alpha proteasome type subunits are considered to play structural roles in the maintenance of the 20S complex as well as mediating the interaction with the 19S regulatory complex (Glickman and Ciechanover, 2002). Protein spots 6207 and 4212 (12 DAA) had higher expression levels in NIL-31, spot 9204 (12 DAA) was a unique protein in NIL-31, and spot 3003 (20 DAA) had a downregulated pattern in NIL-31. These DEPs indicate the presence of different 20S complex types in CS and NIL-31.

Furthermore, the 20S complex cannot hydrolyze proteins on its own, but instead it cleaves them into small peptides and unfolded proteins. These substrates are dominated by key regulatory and signaling proteins with essential roles in cell cycle progression and cellular growth control (Ben-Nissan and Sharon, 2014). In this study, we identified nine DEPs involved with cell development, three of which were related to cellular differentiation and they were found only in NIL-31, i.e., spots 0712, 4810, and 4414 (6 DAA). This suggests that *TaGW2-6A* has a role in the regulation of cell differentiation, and thus the *TaGW2-6A* allelic variants exhibited different patterns due to changes in the structure of the 20S complex.

In addition, the 20S complex is the core particle in the 26S proteasome (Glickman and Ciechanover, 2002; Ben-Nissan and Sharon, 2014). The 26S proteasome is capable of recognizing the polyubiquitin chain and degrading polyubiquitinated proteins into small peptides (Li and Li, 2014). In all eukaryotes, the 26S proteasome pathway plays a key role in regulating diverse aspects of developmental and physiological responses by selectively removing intracellular proteins as well as maintaining the levels of essential proteins for correct biological functioning (Yan et al., 2013). In the present study, the mutant protein might have maintained the 26S proteasome at a suitable level to allow its correct biological functioning.

We identified spot 4211 (6 DAA) as the gibberellin (GA) receptor GID1L2 and its expression level was higher in NIL-31. Our results agreed with those of a previous study, which showed that higher expression of the GA receptor is related to improved biomass production (Li et al., 2013b).

E3 ubiquitin ligases in the UPS are known to have critical roles in hormone perception and signal transduction (Capron et al., 2012). GA is an essential hormone that regulates growth and development in plants, including stem and root elongation, seed germination, floral development, as well as leaf size and shape determination (Fleet and Sun, 2005). GA is an endogenous growth regulator in higher plants and its signaling pathway involves feedback regulation (Griffiths et al., 2006). The DELLA proteins are central to GA signaling, where they repress growth of the style and stigma in the gynoecium (Fuentes et al., 2012). GA signal transduction occurs mainly by promoting DELLA protein degradation (Fuentes et al., 2012) and this metabolic process depends on the UPS. First, GA binds to its receptor to form a GA-receptor complex, which subsequently binds to DELLA proteins tagged with ubiquitin for degradation by the 26S proteasome (Griffiths et al., 2006).

The mutant TaGW2-6A protein could relieve the suppression of GA-dependent growth processes by DELLA proteins to enhance the efficiency of the 26S proteasome in NIL-31. In addition, the greater abundance of GA might promote seed germination and delay leaf senescence, which is consistent with the results of a previous study where *TaGW2-6A* was associated with extending the duration of the green canopy (Simmonds et al., 2014). This might indicate that TaGW2-6A can regulate GA signal transduction and that the mutant TaGW2-6A protein may improve the level of GA signal transduction.

The plant cell wall is the primary site for signal perception and defensive responses to environmental stress (Krzeslowska, 2011). Plants cannot avoid stressful conditions in metal-contaminated areas, such as those affected by cadmium and copper.

Among the DEPs, the main enriched proteins were related to responses to cadmium, and more than one-third of the proteins were related to the copper ion. Cadmium is a toxic, non-essential element for plants, which is harmful to plant cells (Harris and Taylor, 2013; Wang et al., 2014). Genotypic variation is the main factor that determines the effects of cadmium accumulation in plants (Clemens et al., 2013; Harris and Taylor, 2013). Therefore, NIL-31 might enhance the tolerance of cadmium due to the insertion genotype, which could relieve the damage caused to plant cells by cadmium during wheat grain development. Copper is an essential micronutrient for plants, which is involved in several proteins or enzymes during physiological metabolic processes (Maksymiec, 1997). However, excess copper can damage the cellular components or cellular transport processes, thereby causing harmful effects in plants (Liu et al., 2014). These processes might be closely related to the regulation of cell differentiation by TaGW2-6A, thereby suggesting that the mutant TaGW2-6A protein can improve metal stress defenses in wheat.

Heat and drought stress have adverse effects on carbon assimilation and starch synthesis (Skylas et al., 2002). High temperatures affect the functioning of enzymes, possibly by causing complete inactivation as well as influencing membrane-linked processes. Heat shock proteins (HSPs) have various stress-protective roles and they determine the thermotolerance of plants (Tasleem-Tahir et al., 2012; Xue et al., 2013). Thermotolerance is very important for plant survival when plants are subjected to potentially lethal high temperatures. In our study, we identified six DEPs as HSPs, i.e., spot 3609 (20 DAA) was upregulated in NIL-31 and three spots, i.e., spots 1710 (6 DAA), 0807 (6 DAA), and 3314 (12 DAA) were unique to NIL-31, whereas the other two protein spots were not found in NIL-31, i.e., spots 2404 (6 DAA) and 1701 (20 DAA).

Recently, it has been reported that RING E3 ubiquitin ligases have major roles in plant responses to environmental stress. Thus, RGLG2, which functions as a RING E3 ligase, interacts with ATERF53 and negatively regulates the plant drought stress response (Cheng et al., 2012). The RING E3 ubiquitin ligase AtAIRP3/LOG2 also participates in the positive regulation of high-salt and drought stress responses (Kim and Kim, 2013). The RING finger E3 ligase, OsHCI1, drives the nuclear export of multiple substrate proteins and its heterogeneous overexpression enhances acquired thermotolerance (Lim et al., 2013). A previous study showed that a *TaGW2-6A* variant can maintain its production levels in different locations due to its high drought resistance and stable yield capacity (Li et al., 2015). Thereby, we suggest that the mutant TaGW2-6A protein may enhance thermotolerance by regulating the abundance of these HSPs. High temperature and drought are the major types of abiotic stress found in northwest China. Thus, the enhancement of stress resistance might help to explain why this variation is stably inherited during wheat breeding (Kou et al., 2015).

L-ascorbate (or vitamin C) is an essential enzyme cofactor during hydroxylation and other reactions as well as being a primary antioxidant in plants and animals, which are the primary sources of vitamin C for humans (De Tullio and Arrigoni, 2004). In plants, L-ascorbate is involved in many processes, including growth, programmed cell death, pathogen responses, hormone responses, flowering, and senescence, as well as protecting against environmental stresses (Linster and Clarke, 2008). It can activate signal transduction processes in response to stresses by maintaining reactive oxygen species at appropriate levels and ensuring that plants have a balanced internal environment (Tripathy and Oelmüller, 2012). We identified five DEPs related to L-ascorbate. Spot 7105 (6 DAA) was downregulated in NIL-31 and two other proteins, spots 3022 and 3417 (12 DAA), were upregulated, while spots 5426 and 3217 (12 DAA) were identified only in NIL-31. These proteins might contribute to higher stress tolerance in NIL-31.

Serpins are likely to participate in a range of biochemical pathways in distinct plant cell types, tissues, and organs to protect cells from oxidative stress, and they are highly expressed during seed maturation where they occur during all developmental stages in various tissues (Roberts and Hejgaard, 2007). Two types of serpins were identified in this study, i.e., spot 4207 (20 DAA) corresponded to serpin-Z1A, and spots 5211 and 4210 (20 DAA) corresponded to serpin-Z1C. Spot 4207 (20 DAA) was upregulated in NIL-31 and spot 5211 (20DAA) was only detected in NIL-31, whereas spot 4210 (20DAA) was not found in NIL-31. This suggests that the mutant TaGW2-6A protein can change the conformation of serpin-Z1C.

Translationally-controlled tumor protein (TCTP) is a multifunctional protein with important roles in immune responses, cell proliferation, tumorigenicity, and cell apoptosis (Gu et al., 2014). In plants, TCTP significantly decreases induced cell death and it reduces plant responses to ethylene as well as promoting plant growth by accelerating cell proliferation (Hoepflinger et al., 2013). The TCTP protein was related to spots 3216 and 0212 (12 DAA) in this study and these spots were more abundant in NIL-31, in which this protein could enhance heat and drought tolerance as well as regulating cell development, thereby indicating that the TaGW2-6A protein can regulate cell differentiation.

Starch is the major component of wheat grain (Tasleem-Tahir et al., 2012) and a major energy reserve in a large variety of higher green plants (Miao et al., 2015). Carbohydrate metabolism enzymes and 14-3-3 proteins play important roles in starch accumulation (Wang et al., 2016). Spot 1103 (6 DAA) was identified as 14-3-3 protein and it had a low expression level in NIL-31. β-amylase has a key role in the hydrolysis of starch and it also function as an osmoprotectant to protect the cell structure, as well as supporting respiration under low photosynthesis conditions and other types of stress by stimulating starch degradation and maltose accumulation (Srivastava and Kayastha, 2013). It may mediate fluctuations in endogenous sugar levels in plants during the diurnal cycle. We identified six spots related to β-amylase, where two spots had contrasting regulatory patterns in NIL-31. The upregulated spot 2603 (12 DAA) might facilitate the balance between sugars in the day and night. The downregulated spot 4511 (12 DAA) could be related to higher stress tolerance due to TaGW2-6A. E3 has a newly defined role in the plant sugar response (Huang et al., 2010) and our results showed that TaGW2-6A might be implicated in this response.

Protein disulfide isomerases play different roles during the maturation of the secreted plasma membrane and storage proteins, as well as being involved in various stress responses (Houston et al., 2005). Their functions are differentiated by structural changes (Zhu et al., 2014). In this study, spots 7314 and 7315 (6 DAA) were found only in NIL-31, in which they might function to accelerate the synthesis of storage proteins.

Growth is underpinned by carbohydrate metabolism (Reinhold et al., 2011), which is tightly linked to photosynthesis in plants and essential for the supply of energy and the carbon skeleton to the entire organism (Kunz et al., 2014). In carbohydrate metabolism, phosphoglucomutases (PGMs) catalyze the reversible interconversion of glucose 6-phosphate and glucose 1-phosphate, where PGMs exist as plastidial (pPGMs) and cytosolic (cPGMs) isoforms. A lack of PGMs reduces the starch levels but there are higher amounts of soluble sugars (Malinova et al., 2014). In addition, the stability of DELLA proteins is considered to be controlled by protein phosphorylation and dephosphorylation (Qin et al., 2014b). In this study, spot 4710 (6 DAA) was an upregulated PGM protein in NIL-31 and the unique PGM spot 6718 (6 DAA) could also lead to greater starch accumulation in NIL-31, which might have reduced the expression level of UTP-glucose-1-phosphate uridylyl transferase protein spot 3409 (12 DAA) in NIL-31 (Thoden and Holden, 2007). Thus, TaGW2-6A might regulate GA metabolic processes by influencing the formation of PGMs.

Glycolysis provides energy and intermediates to facilitate the synthesis of metabolites. In our study, the main type of protein related to glycolysis was phosphoglycerate kinase, which participates in the glycolytic, gluconeogenic, and photosynthetic pathways. iPGM catalyzes an intramolecular reaction that requires a divalent metal cation for its activity, such as Mn^2+^ or Co^2+^ (Collet et al., 2001). iPGMs are key enzymes that catalyze the interconversion of 2- and 3-phosphoglycerate in the glycolytic and gluconeogenic pathways, which are present in the majority of cellular organisms (Singh et al., 2013, 2014). Among iPGM proteins, protein spot 4601 (6 DAA) had a high expression level in NIL-31, whereas protein spots 4608 (12 DAA), and 4603 and 4604 (20 DAA) were downregulated in NIL-31, which suggests a greater capacity for metal binding during early development in NIL-31.

The TCA cycle is a central pathway for the metabolism of sugars, lipids, and amino acids. Isocitrate dehydrogenase (ICDH) is an ideal candidate enzyme for starting physiological studies of the C/N balance in the incomplete TCA cycle due to its position at the branching point between carbon and nitrogen metabolism pathways. In addition, ICDH can catalyze the oxidative decarboxylation of isocitrate to produce 2-oxoglutarate (2-OG) (Martin et al., 2014). In this study, spot 6405 (6 DAA) was identified as ICDH and it was upregulated in NIL-31, possibly resulting in a higher abundance of 2-OG. Most of the 2-OG can be transformed into succinic semialdehyde and then into succinate (Martin et al., 2014). Spot 5711 (6 DAA) was identified as succinate dehydrogenase and it was detected only in NIL-31, which might be related to the ICDH protein. Succinate dehydrogenase has important functions in both the TCA cycle and aerobic respiratory chain, as well as being related to an enhanced rate of photosynthesis (Araujo et al., 2011). These results might help to understand a previous report that the insertion type of *TaGW2-6A* has a higher capacity for capturing light energy, which is converted into biomass (Li et al., 2015).

## Conclusion

In this study, we confirmed that *TaGW2-6A* is a negative regulator of grain size and weight. The TaGW2-6A protein may play key roles during the regulation of cell differentiation and grain filling during grain development. We found that the mutant TaGW2-6A protein could enhance wheat stress tolerance and facilitate GA signal metabolism. Our results provide new insights into the genetic mechanisms regulated by *TaGW2-6A* as well as improving our understanding of the biological processes involved in seed formation.

## Author contributions

XL designed the project and amended the manuscript. DD and XG performed the experiments, analyzed the data, and drafted the manuscript. JG, QYL, and QL collected the data. LL assisted with the technical experiments. All of the authors read and approved the manuscript.

### Conflict of interest statement

The authors declare that the research was conducted in the absence of any commercial or financial relationships that could be construed as a potential conflict of interest.

## Supplementary material

Table S1 **Comparison of the ***TaGW2-6A*** sequences from Chinese Spring, NIL-31, and Lankaodali**.

Table S2 **Alignment of the predicted amino acid sequences of the TaGW2-6A protein from Chinese Spring, NIL-31, and Lankaodali**.

Table S3 **Standard curve obtained using the Bradford assay**.

Table S4 **Differentially expressed proteins identified by MALDI-TOF/TOF-MS at three grain developmental stages in Chinese Spring and NIL-31**.

Table S5 **GO and KEGG analysis of the differentially expressed proteins in Chinese Spring and NIL-31**.

Table S6 **GO and KEGG analysis of the differentially expressed proteins**.

Data Sheet S1 **Results of MALDI-TOF/TOF-MS analysis**.

Data Sheet S2 **Results of GO and KEEG analyses**.
