# Supplementary material for: Identification of Key Proteins and Networks Related to Grain Development in Wheat (Triticum aestivum L.) by Comparative Transcription and Proteomic Analysis of Allelic Variants in TaGW2-6A
Source: Front Plant Sci. 2016 Jun 28;7:922. doi: 10.3389/fpls.2016.00922 (PMC4923154; doi:10.3389/fpls.2016.00922)
Supplement: Table S1 — Comparison of the TaGW2-6A sequences from Chinese Spring, NIL-31, and Lankaodali. [file Table1.PDF]

Identification of key proteins and networks related to grain development in wheat (*Triticum aestivum* L.) by comparative proteomic analysis of allelic variants in *TaGW2-6A*

Dengfeng Du†, Xin Gao†, Juan Geng, Qingyan Li, Liqun Li, Qian Lv, Xuejun Li*

State Key Laboratory of Crop Stress Biology in Arid Areas and College of Agronomy, Northwest A&F University, Yangling, Shaanxi 712100, People's Republic of China.

*** Correspondence:** Northwest A&F University, 3 Taicheng Rd, Yangling, Shaanxi Province 712100, People's Republic of China;

Telephone (Fax): +86 29 8708 2022; Email: xuejun@nwsuaf.edu.cn

†These authors contributed equally to this work.

Supplementary Table S1. Comparison of the *TaGW2-6A* sequences from Chinese Spring (*TaGW2-6A-CS*), NIL-31 (*TaGW2-6A-NIL-31*) and Lankaodali (*TaGW2-6A-LK*). The T-base insertion site is indicated by an asterisk.

MSF: 1276 Type: P Check: 1726

Name: *TaGW2-6A-CS* oo Len: 1276 Check: 3321 Weight: 1.0

Name: *TaGW2-6A-NIL-31* oo Len: 1276 Check: 4220 Weight: 1.0

Name: *TaGW2-6A-LK* oo Len: 1276 Check: 4185 Weight: 1.0

TaGW2-6A-CS	1	ATGGGGAACAGAATAGGGGGGAGGAGGAAGGCCGGGGTGGAGGAGCGGTA
TaGW2-6A-NIL-31	1	ATGGGGAACAGAATAGGAGGGAGGAGGAAGGCCGGGGTGGAGGAGCGGTA
TaGW2-6A-LK	1	ATGGGGAACAGAATAGGAGGGAGGAGGAAGGCCGGGGTGGAGGAGCGGTA
TaGW2-6A-CS	51	CACGAGGCCGCAGGGGCTGTACGAGCACAGGGATATCGACCAGAAGAAGC
TaGW2-6A-NIL-31	51	CACGAGGCCGCAGGGGCTGTACGAGCACAGGGATATCGACCAGAAGAAGC
TaGW2-6A-LK	51	CACGAGGCCGCAGGGGCTGTACGAGCACAGGGATATCGACCAGAAGAAGC

33	*TaGW2-6A-CS*	101	TACGCAAGTTGATCCTCGAGGCCAAACTCGCGCCCTGCTACCCGGGGGCT
34	*TaGW2-6A-NIL-31*	101	TACGCAAGTTGATCCTCGAGGCCAAGCTCGCGCCCTGCTACCCGGGGGCT
35	*TaGW2-6A-LK*	101	TACGCAAGTTGATCCTCGAGGCCAAGCTCGCGCCCTGCTACCCGGGGGCT
36			
37	*TaGW2-6A-CS*	151	GACGACGCCGCGGGGGGTGACCTGGAGGAGTGCCCCATCTGCTTCCTGTA
38	*TaGW2-6A-NIL-31*	151	GACGACGCCGCGGGGGGTGACCTGGAGGAGTGCCCCATCTGCTTCCTGTA
39	*TaGW2-6A-LK*	151	GACGACGCCGCGGGGGGTGACCTGGAGGAGTGCCCCATCTGCTTCCTGTA
40			
41	*TaGW2-6A-CS*	201	CTACCCAAGCCTTAACCGATCAAAATGTTGCTCGAAAGGGATATGTACAG
42	*TaGW2-6A-NIL-31*	201	CTACCCAAGCCTTAACCGATCAAAATGTTGCTCGAAAGGGATATGTACAG
43	*TaGW2-6A-LK*	201	CTACCCAAGCCTTAACCGATCAAAATGTTGCTCGAAAGGGATATGTACAG
44			
45	*TaGW2-6A-CS*	251	AGTGCTTTCTTCAAATGAAACCAACTCATACTGCTCGACCTACACAATGC
46	*TaGW2-6A-NIL-31*	251	AGTGCTTTCTTCAAATGAAACCAACTCATACTGCTCGACCTACACAATGC
47	*TaGW2-6A-LK*	251	AGTGCTTTCTTCAAATGAAACCAACTCATACTGCTCGACCTACACAATGC
48			
49	*TaGW2-6A-CS*	301	CCATTCTGCAAAACCCCCAACTATGCTGTGGAGTATCGTGGTGTAAAGAC
50	*TaGW2-6A-NIL-31*	301	CCATTCTGCAAAACCCCCAACTATGCTGTGGAGTATCGTGGTGTAAAGAC
51	*TaGW2-6A-LK*	301	CCATTCTGCAAAACCCCCAACTATGCTGTGGAGTATCGTGGTGTAAAGAC
52			
53	*TaGW2-6A-CS*	351	AAAGGAGGAAAGGAGCATAGAGCAATTTGAAGAACAGAAAGTCATTGAAG
54	*TaGW2-6A-NIL-31*	351	AAAGGAGGAAAGGAGCATAGAGCAATTTGAAGAACAGAAAGTCATTGAAG
55	*TaGW2-6A-LK*	351	AAAGGAGGAAAGGAGCATAGAGCAATTTGAAGAACAGAAAGTCATTGAAG
56			
57	*TaGW2-6A-CS*	401	CACAGATGAGGGTGCGGCAGCAAGCACTTCAAGACGAAGAGGATAAGGTG
58	*TaGW2-6A-NIL-31*	401	CACAGATGAGGGTGCGGCAGCAAGCACTTCAAGACGAAGAGGATAAGATG
59	*TaGW2-6A-LK*	401	CACAGATGAGGATGCGCCAGCAAGCACTTCAAGACGAAGAGGATAAGATG
60			
61			
62			

63	*TaGW2-6A-CS*	451	AAAAGAAAACAGAGTAGGTGCTCTTCTAGCAGAACAATCGCTCCAACAAC
64	*TaGW2-6A-NIL-31*	451	AAAAGAAAACAGAGTAGGTGCTCTTCTAGCAGAACAATCGCTCCAACAAC
65	*TaGW2-6A-LK*	451	AAAAGAAAACAGAGTAGGTGCTCTTCTAGCAAACAATCGCCTCCAACAAC
66			
67	*TaGW2-6A-CS*	501	AGAAGTGGAGTATCGAGATATTTGCAGCACATCCTATTCAGTGCCATCGT
68	*TaGW2-6A-NIL-31*	501	AGAAGTGGAGTATCGAGATATTTGCAGCACATCCTATTCAGTGCCATCGT
69	*TaGW2-6A-LK*	501	AGAAGTGGAGTATCGAGATATTTGCAGCACATCCTATTCAGTGCCATCGT
70			
71	*TaGW2-6A-CS*	551	ACCAATGTACCCAGCAAGAAACTGAATGTTGTTCGTCTGAGCCTTCATGT
72	*TaGW2-6A-NIL-31*	551	ACCAATGTACCCAGCAAGAAACTGAATGTTGTTCGTCTGAGCCTTCATGT
73	*TaGW2-6A-LK*	551	ACCAATGTACCGAGCAAGAAACTGAATGTTGTTCATCTGAGCCTTCATGT
74			
75	*TaGW2-6A-CS*	601	TCTGCTCAGGCTAACATGCGGTCTTTCCATTCTAGGCATACTCGTGATGA
76	*TaGW2-6A-NIL-31*	601	TCTGCTCAGGCTAACATGCGGTCTTTCCATTCTAGGCATACTCGTGATGA
77	*TaGW2-6A-LK*	601	TCTGCTCAGGCTAACATGCGGTCTTTCCATTCTAGGCATACTCGTGATGA
78			
79	*TaGW2-6A-CS*	651	TAACATAGACATGAACATAGAGGACATGATGGTTATGGAAGCGATTTGGC
80	*TaGW2-6A-NIL-31*	651	TAACATAGACATGAACATAGAGGACATGATGGTTATGGAAGCGATTTGGC
81	*TaGW2-6A-LK*	651	TAACATAGACATGAACATAGAGGACATGATGGTTATGGAAGCGATTTGGC
82			
83	*TaGW2-6A-CS*	701	GTTCAATTCAGGAGCAAGGAAGTATAGGAAATCCTTCTTGTGGGAGCTTT
84	*TaGW2-6A-NIL-31*	701	GTTCAATTCAGGAGCAAGGAAGTATAGGAAATCCTTCTTGTGGGAGCTTT
85	*TaGW2-6A-LK*	701	GTTCAATTCAGGAGCAAGGAAGTATAGGAAATCCTGCTTGTGGGAGTTTT
86			
87	*TaGW2-6A-CS*	751	ATGCCTTTTGAGCAACCAACGCGTGAGAGGCAGGCATTCGTTGCAGCTCC
88	*TaGW2-6A-NIL-31*	751	ATGCCTTTTGAGCAACCAACGCGTGAGAGGCAGGCATTCGTTGCAGCTCC
89	*TaGW2-6A-LK*	751	ATGCCTTTTGAGCAACCAACGCGTGAGAGGCAGGCATTCGTTGCAGCTCC
90			
91			
92			

```
93   TaGW2-6A-CS              801   TCCTCTAGAAATGCCCCATCCTGGTGGATTTTCTTGTGCTGTTGCTGCTA
94   TaGW2-6A-NIL-31          801   TCCTCTAGAAATGCCCCATCCTGGTGGATTTTCTTGTGCTGTTGCTGCTA
95   TaGW2-6A-LK              801   TCCTCTAGAAATGCCCCATCCTGGTGGATTTTCTTGTGCTGTTGCTGCTA
96
97   TaGW2-6A-CS              851   TGGCTGAGCACCAGCCATCAAGCATGGATTTCTCTTACATGACTGGTAGT
98   TaGW2-6A-NIL-31          851   TGGCTGAGCACCAGCCATCAAGCATGGATTTCTCTTACATGACTGGTAGT
99   TaGW2-6A-LK              851   TGGCTGAGCACCAGCCATCAAGCATGGATTTCTCTTACATGACTGGTAGT
100
101  TaGW2-6A-CS              901   AGTGCGTTCCCAGTCTTTGACATGTTCCGCCGACCGTGCAACATTGCTGG
102  TaGW2-6A-NIL-31          901   AGTGCGTTCCCAGTCTTTGACATGTTCCGCCGACCGTGCAACATTGCTGG
103  TaGW2-6A-LK              901   AGTGCGTTCCCAGTCTTTGACATGTTCCGCCGACCGTGCAACATTGCTGG
104                                                  *
105  TaGW2-6A-CS              951   TGGAAGCATGGGTGCTGCGGAAAGTT-CACCAGATAGCTGGAGCGGGATA
106  TaGW2-6A-NIL-31          951   TGGAAGCATGGGTGCTGCGGAAAGTTTCACCAGATAG--------------
107  TaGW2-6A-LK              951   TGGAAGCATGGGTGCTGCGGAAAGTTTCACCAGATAG--------------
108
109  TaGW2-6A-CS             1001   GCGCCAAGTTGCAGCAGAAGGGAAGTGGTAAGAGAGGAAGGAGAGTGCTC
110  TaGW2-6A-NIL-31                ------------------------------------------------
111  TaGW2-6A-LK                    ------------------------------------------------
112
113  TaGW2-6A-CS             1051   AACCGACCACTTGTCAGAGGGTGCAGAGGCCGGGACAAGCTATGCCGGCT
114  TaGW2-6A-NIL-31                -----------------------------------------------
115  TaGW2-6A-LK                    -----------------------------------------------
116
117  TaGW2-6A-CS             1101   CGGACATTGTGGTGGATGCGGGGACAATGCTACCGTTGCCTTTTGCTGAC
118  TaGW2-6A-NIL-31                ------------------------------------------------
119  TaGW2-6A-LK                    ------------------------------------------------
120
121
122
```

123 *TaGW2-6A-CS* 1151 AATTACAGTATGGTTGCAAGCCATTTCCGTCCTGAGAGCATCGAAGAACA

124 *TaGW2-6A-NIL-31* ----------

125 *TaGW2-6A-LK* ----------

126

127 *TaGW2-6A-CS* 1201 AATGATGTATTCCATGGCTGTTTCTTTAGCAGAAGCTCATGGTAGAACGC

128 *TaGW2-6A-NIL-31* ----------

129 *TaGW2-6A-LK* ----------

130

131 *TaGW2-6A-CS* 1251 ACACGCAAGGGTTGGCATGGTTGT--

132 *TaGW2-6A-NIL-31* ----------

133 *TaGW2-6A-LK* ----------
