# Supplementary material for: Identification of Key Proteins and Networks Related to Grain Development in Wheat (Triticum aestivum L.) by Comparative Transcription and Proteomic Analysis of Allelic Variants in TaGW2-6A
Source: Front Plant Sci. 2016 Jun 28;7:922. doi: 10.3389/fpls.2016.00922 (PMC4923154; doi:10.3389/fpls.2016.00922)
Supplement: Table S2 — Alignment of the predicted amino acid sequences of the TaGW2-6A protein from Chinese Spring, NIL-31, and Lankaodali. [file Table2.PDF]

Identification of key proteins and networks related to grain development in wheat (*Triticum aestivum* L.) by comparative proteomic analysis of allelic variants in *TaGW2-6A*

Dengfeng Du[†], Xin Gao[†], Juan Geng, Qingyan Li, Liqun Li, Qian Lv, Xuejun Li*

State Key Laboratory of Crop Stress Biology in Arid Areas and College of Agronomy, Northwest A&F University, Yangling, Shaanxi 712100, People's Republic of China.

*** Correspondence:** Northwest A&F University, 3 Taicheng Rd, Yangling, Shaanxi Province 712100, People's Republic of China;

Telephone (Fax): +86 29 8708 2022; Email: xuejun@nwsuaf.edu.cn

[†]These authors contributed equally to this work.

Supplementary Table S2. Alignment of predicted amino acid sequence of the TaGW2-6A protein from CS,NIL-31 and Lankaodali, the RING-domain were *boxed,* altered amino acids at the SNP sites were also displayed.

MSF: 424 Type: N Check: 6554

Name: TaGW2-6A-CS oo Len: 424 Check: 457 Weight: 1.0

Name: TaGW2-6A-NIL-31 oo Len: 424 Check: 7576 Weight: 1.0

Name: TaGW2-6A-LK oo Len: 424 Check: 8521 Weight: 1.0

TaGW2-6A-CS	1	MGNRIGGRRKAGVEERYTRPQGLYEHRDIDQKKLRKLILEAKLAPCYPGADDAAGGDLEE
TaGW2-6A-NIL-31	1	MGNRIGGRRKAGVEERYTRPQGLYEHRDIDQKKLRKLILEAKLAPCYPGADDAAGGDLEE
TaGW2-6A-LK	1	MGNRIGGRRKAGVEERYTRPQGLYEHRDIDQKKLRKLILEAKLAPCYPGADDAAGGDLEE

```
TaGW2-6A-CS          61   CPICFLYYPSLNRSKCCSKGICTECFLQMKPTHTARPTQCPFCKTPNYAVEYRGVKTKEE
TaGW2-6A-NIL-31      61   CPICFLYYPSLNRSKCCSKGICTECFLQMKPTHTARPTQCPFCKTPNYAVEYRGVKTKEE
TaGW2-6A-LK          61   CPICFLYYPSLNRSKCCSKGICTECFLQMKPTHTARPTQCPFCKTPNYAVEYRGVKTKEE

TaGW2-6A-CS         121   RSIEQFEEQKVIEAQMRVRQQALQDEEDKVKRKQSRCSSSRTIAPTTEVEYRDICSTSYS
TaGW2-6A-NIL-31     121   RSIEQFEEQKVIEAQMRVRQQALQDEEDKMKRKQSRCSSSRTIAPTTEVEYRDICSTSYS
TaGW2-6A-LK         121   RSIEQFEEQKVIEAQMRMRQQALQDEEDKMKRKQSRCSSSRTIAPTTEVEYRDICSTSYS

TaGW2-6A-CS         181   VPSYQCTQQETECCSSEPSCSAQANMRSFHSRHTRDDNIDMNIEDMMVMEAIWRSIQEQG
TaGW2-6A-NIL-31     181   VPSYQCTQQETECCSSEPSCSAQANMRSFHSRHTRDDNIDMNIEDMMVMEAIWRSIQEQG
TaGW2-6A-LK         181   VPSYQCTEQETECCSSEPSCSAQANMRSFHSRHTRDDNIDMNIEDMMVMEAIWRSIQEQG

TaGW2-6A-CS         241   SIGNPSCGSFMPFEQPTRERQAFVAAPPLEMPHPGGFSCAVAAMAEHQPSSMDFSYMTGS
TaGW2-6A-NIL-31     241   SIGNPSCGSFMPFEQPTRERQAFVAAPPLEMPHPGGFSCAVAAMAEHQPSSMDFSYMTGS
TaGW2-6A-LK         241   SIGNPACGSFMPFEQPTRERQAFVAAPPLEMPHPGGFSCAVAAMAEHQPSSMDFSYMTGS

TaGW2-6A-CS         301   SAFPVFDMFRRPCNIAGGSMGAAESSPDSWSGIAPSCSRREVVREEGECSTDHLSEGAEA
TaGW2-6A-NIL-31     301   SAFPVFDMFRRPCNIAGGSMGAAESFTR--------------------------------
TaGW2-6A-LK         301   SAFPVFDMFRRPCNIAGGSMGAAESFTR--------------------------------

TaGW2-6A-CS         361   GTSYAGSDIVVDAGTMLPLPFADNYSMVASHFRPESIEEQMMYSMAVSLAEAHGRTHTQG
TaGW2-6A-NIL-31           ------------------------------------------------------------
```

TaGW2-6A-LK		--
TaGW2-6A-CS	421	LAWL
TaGW2-6A-NIL-31		----
TaGW2-6A-LK		----
